# Supplementary material for: A Re‐Appraisal of Three Network Meta‐Analyses to Explain the Discrepancy in Findings for the Efficacy of Fluoxetine for the Treatment of Depression in Children and Adolescents
Source: Cochrane Evid Synth Methods. 2026 Oct 1;4(6):e70108. doi: 10.1002/cesm.70108 (PMC13628150; doi:10.1002/cesm.70108)
Supplement: Supplementary file 1 [file CESM-4-e70108-s001.pdf]

Appendix

A Re-Appraisal of Three Network Meta-Analyses to Explain the Discrepancy in Findings for the Efficacy of Fluoxetine for the Treatment of Depression in Children and Adolescents

Figure S1. Standardized mean difference (SMD) of Attari et al. 2006, based on the pre- and post measures reported in their Table 2 in the original article, and an assumed correlation between pre- and post-measures between 0 and 0.99. An SMD of 4.22 (dashed horizontal line) can only be achieved with nearly perfect correlation r > 0.97 (dashed vertical line).

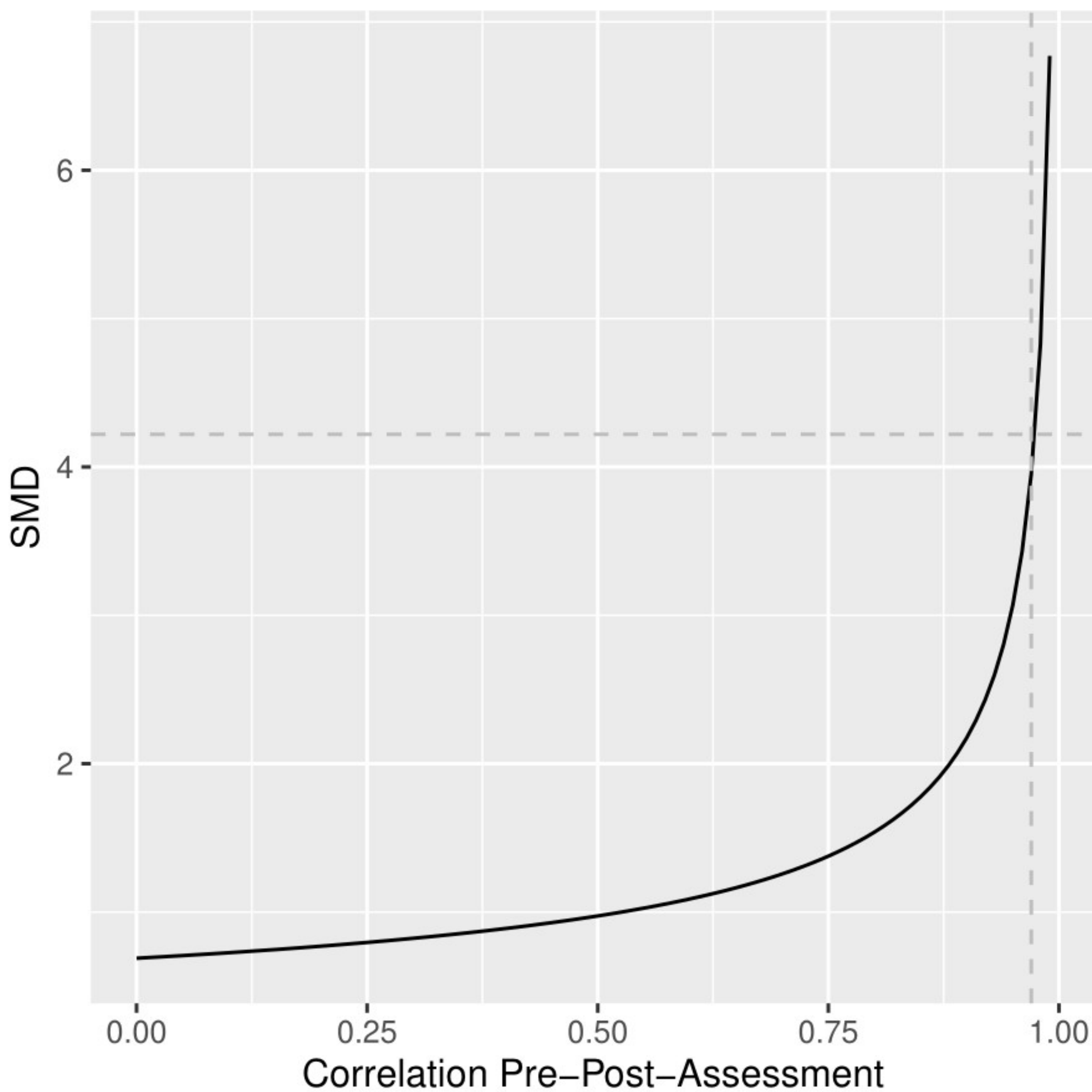

Figure S2. Outlier-analyses of Cipriani et al. (2016), Attari et al. (2006) is study #4.

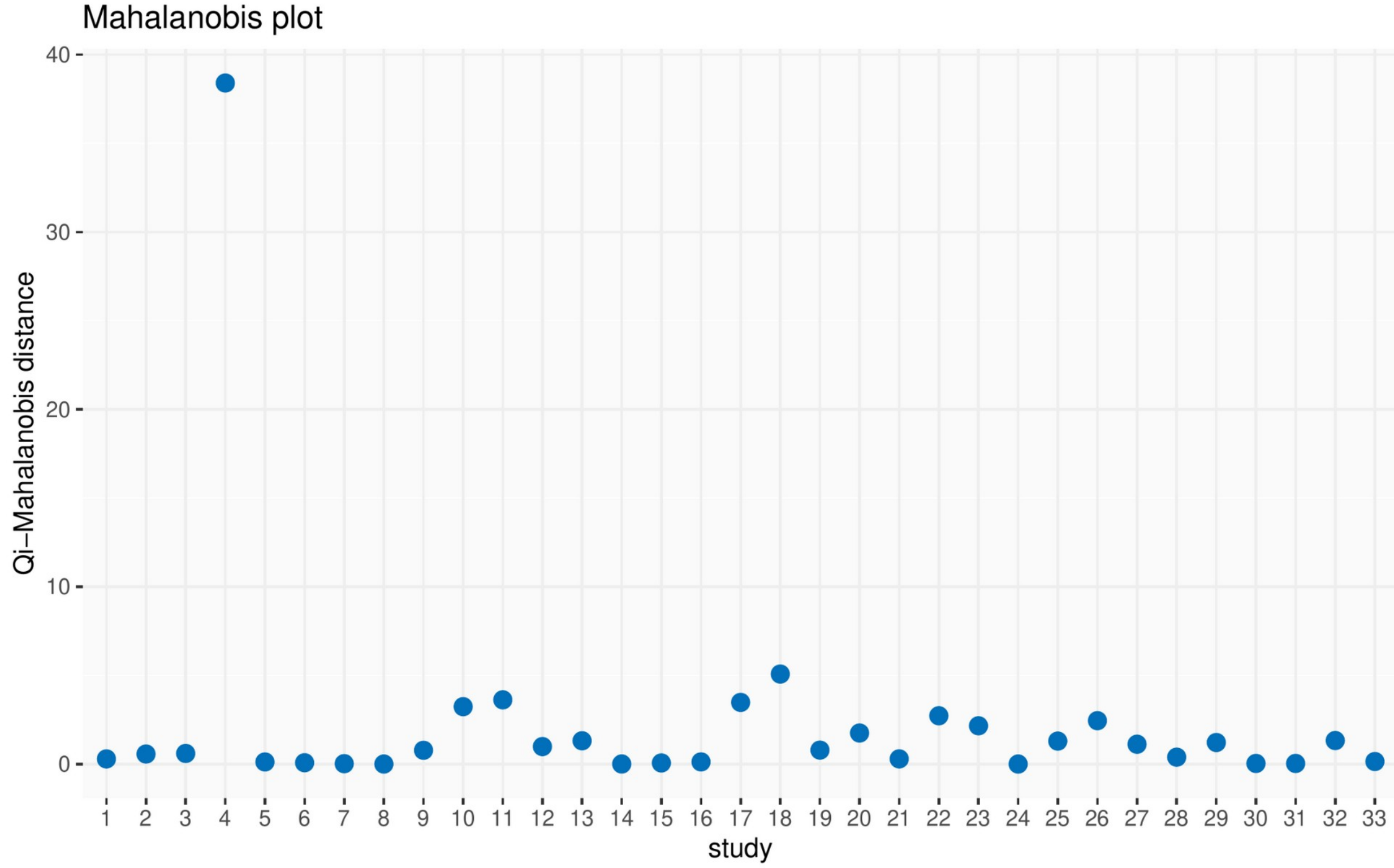

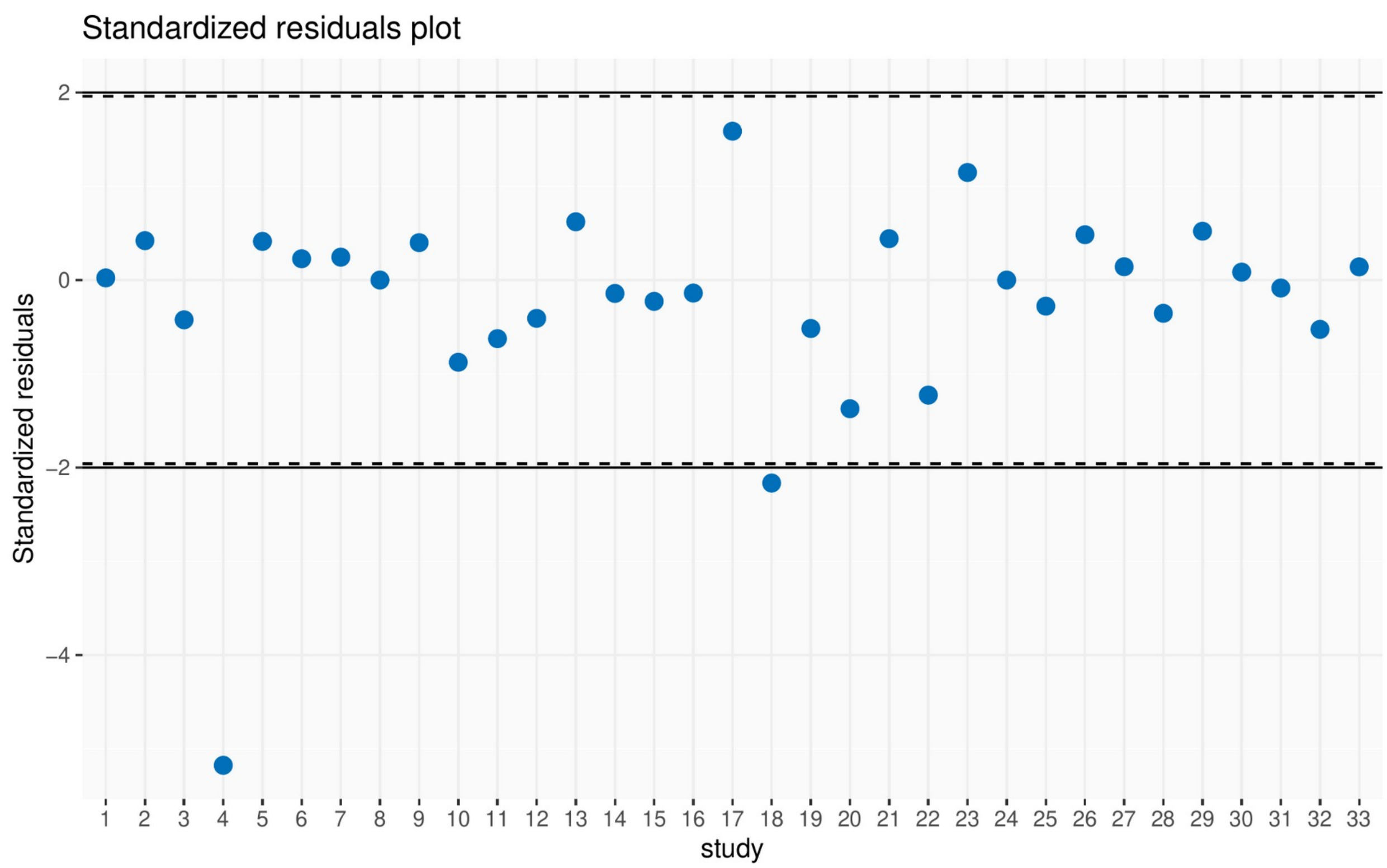

Figure S3. Outlier-analyses of Zhou, Attari et al. (2006) is study #13.

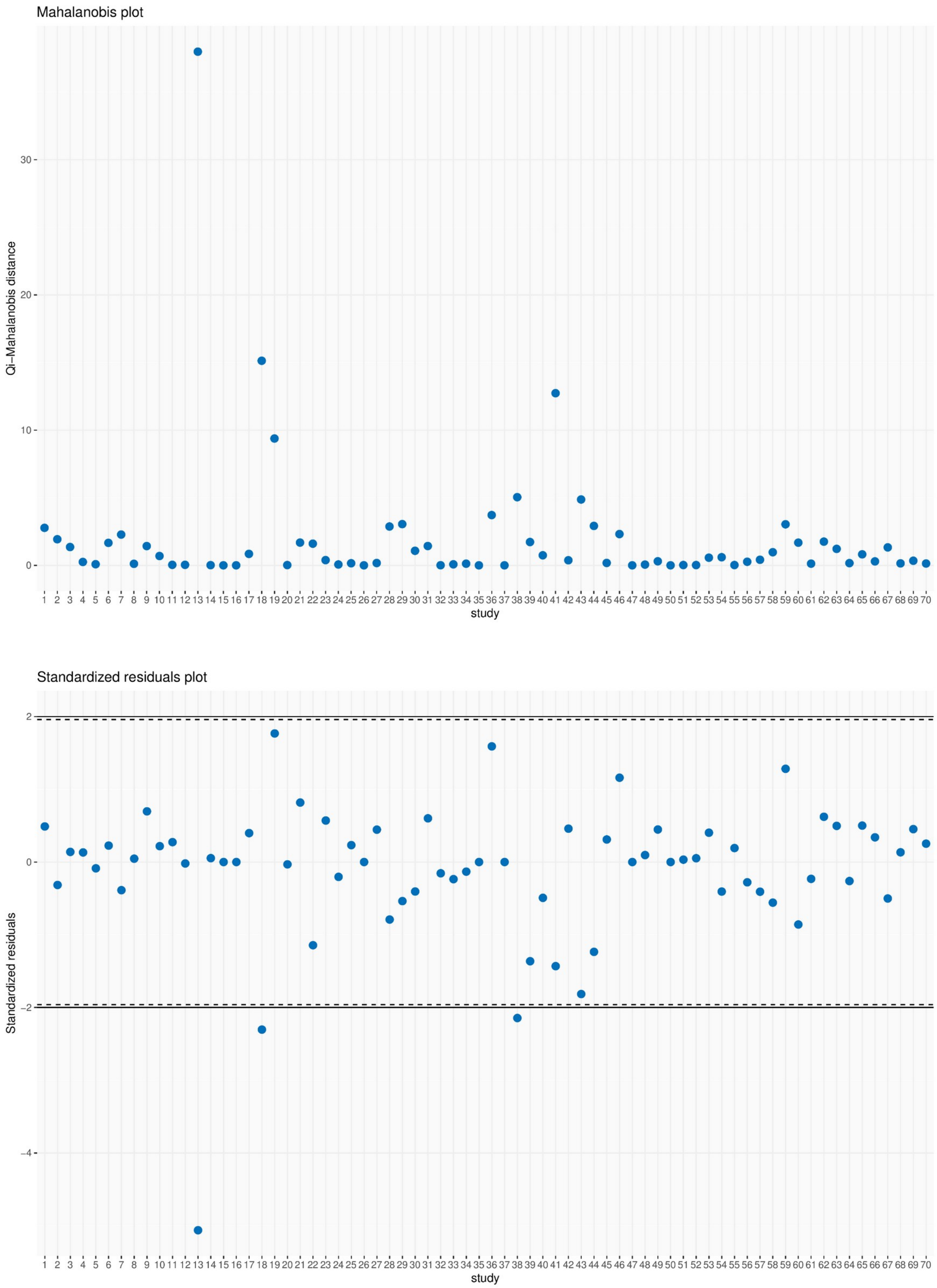

Table S1. Results of our sensitivity analyses using a frequentist approach instead of a Bayesian approach for fluoxetine efficacy versus placebo with and without Attari et al. (2006) using the Cipriani et al. and Zhou et al. data.

**Study**	**Analysis**	**Direct SMD (95% CI)**	**Indirect SMD (95% CI)**	**p-value for the test of incoherence**	**Combined SMD (95% CI)**
Cipriani 2016	With Attari	-0.26 (-0.51 to -0.01)	-1.37 (-2.12 to -0.63)	0.0057	-0.37 (-0.61 to -0.14)
	Without Attari	-0.27 (-0.43 to -0.10)	-0.15 (-0.81 to 0.51)	0.7162	-0.26 (-0.43 to -0.10)
Zhou 2020	With Attari	-0.24 (-0.48 to 0.00)	-1.32 (-1.91 to -0.72)	0.0010	-0.39 (-0.62 to -0.17)
	Without Attari	-0.24 (-0.45 to -0.04)	-0.67 (-1.24 to -0.10)	0.1669	-0.29 (-0.49 to -0.10)
